# L'otite moyenne chronique cholestéatomateuse de l'enfant: à propos de 30 cas

**DOI:** 10.11604/pamj.2015.21.24.5689

**Published:** 2015-05-08

**Authors:** Mohamed Mliha Touati, Youssef Darouassi, Mehdi Chihani, Brahim Bouaity, Haddou Ammar

**Affiliations:** 1 Service d'Oto-rhino-laryngologie et Chirurgie Cervico-faciale, Hôpital Militaire Avicenne, Marrakech, Maroc

**Keywords:** otite moyenne chronique cholestéatomateuse, enfant, tympanoplastie en technique fermée, cholesteatomatous chronic otitis media, child, closed tympanoplasty

## Abstract

Le cholstéatome de l'oreille moyenne est une otite chronique qualifiée de dangereuse en raison de ses risques évolutifs et de ses complications potentiellement graves pouvant mettre en jeu le pronostic vital. L’évolution du cholestèatome est sensiblement différente entre l'adulte et l'enfant, avec une agressivité particulière des cholestèatomes pédiatriques nécessitant ainsi une prise en charge adéquate. Le but de notre travail est d'analyser à travers une revue bibliographique les particularités des otites moyennes chroniques cholestèatomateuses chez 30 enfants pris en charge dans le service d'oto-rhino-laryngologie de l'hôpital militaire Avicenne de Marrakech. Le traitement est exclusivement chirurgical, actuellement, la plupart des auteurs optent pour l'utilisation d'une technique fermée en première intention chez l'enfant

## Introduction

Le cholstéatome de l'oreille moyenne est une otite chronique qualifiée de dangereuse en raison de ses risques évolutifs et de ses complications potentiellement graves, justifiant pleinement le recours exclusif à un traitement chirurgical [1]. Chez l'enfant, le cholestéatome présente une plus grande agressivité, responsable d'une extension importante et d'un taux plus élevé de cholestéatomes résiduels et de récidives [2]. Le traitement est exclusivement chirurgical, actuellement, la plupart des auteurs optent pour l'utilisation d'une technique fermée en première intention chez l'enfant [1, 2].

## Méthodes

C'est une étude rétrospective effectuée au service d'ORL et CCF de l'Hôpital Militaire Avicenne de Marrakech portant sur 30 enfants âgés de moins de 15 ans présentant une otite moyenne chronique cholestéatomateuse, cette étude s’étale sur une période de cinq ans (janvier 2009 à décembre 2013). Le but de notre travail est d'analyser à travers une large revue bibliographique les particularités épidémiologiques, cliniques, paracliniques, thérapeutiques et évolutives des otites moyennes chroniques cholestèatomateuses chez 30 enfants pris en charge dans le service d'oto-rhino-laryngologie de l'hôpital militaire Avicenne de Marrakech.

## Résultats

**Figure 1:**
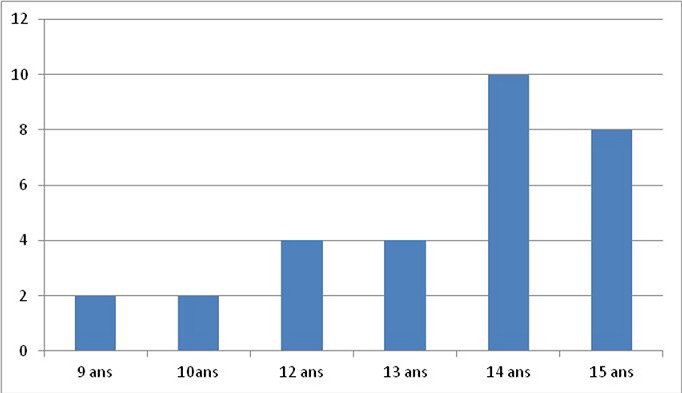
Répartition des enfants selon l’âge

**Figure 2:**
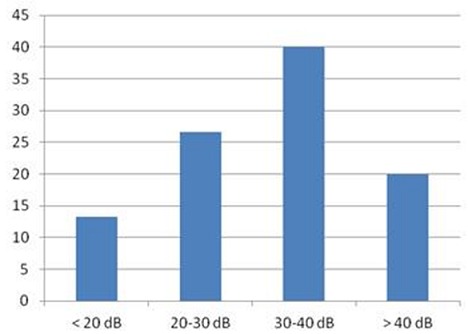
Rinne audiométrique moyen en préopératoire

**Figure 3:**
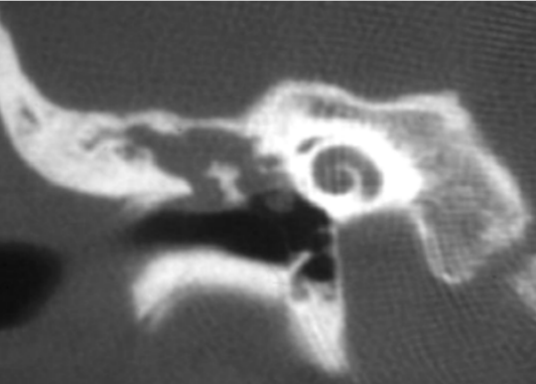
TDM de l'oreille moyenne en coupe coronale montrant un cholestéatome attical avec lyse du mur de la logette

**Figure 4:**
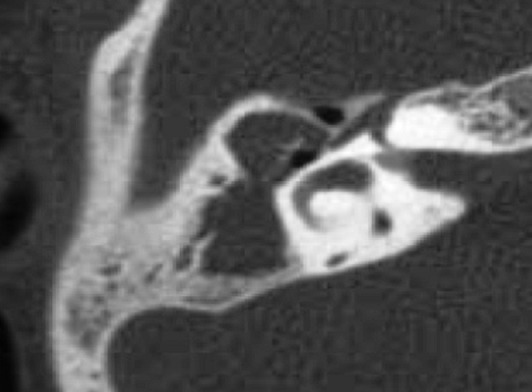
TDM de l'oreille moyenne en coupe axiale montrant un cholestéatome antro-attical avec lyse de la chaîne ossiculaire

L’âge moyen de nos patients était de 13.26 ans, avec des extrêmes allant de 09 à 15ans (Figure 1), il s'agissait de 9 garçons et 6 filles soit un sex-ratio de 1,5.24 enfants, soit 80% présentaient des otites à répétition, on n'a pas noté la notion de traumatisme auriculaire ni de chirurgie otologique. Le délai moyen de consultation était de 10 mois, l'oreille droite était touchée dans 53.3% des cas, l'oreille gauche dans 40% des cas, une atteinte bilatérale a été notée dans 2 cas, soit 6,6%. Les principaux signes cliniques qui ont incités les patients à consulter étaient dominés par une otorrhée chronique purulente et fétide retrouvée dans 24 cas, soit 80%, l'association hypoacousie-otorrhée dans 18 cas soit 60%, aucune complication révélatrice du cholestéatome n'a été rapportée. Cliniquement, une perforation tympanique postéro-supérieure et marginale était observée dans 20 cas (66,6%), Une poche de rétraction dans 8 cas soit 26, 6% et un polype attical dans 2 cas, soit 6,6%. Sur le plan paraclinique, l'audiométrie tonale liminaire réalisée en préopératoire pour tous les enfants, avait montré Une surdité de transmission pure dans 86,6% des cas, avec un Rinne audiométrique moyen de 30 a 40 dB dans 40% des cas (Figure 2). Une surdité mixte dans 13.3%, et 2 cas surdité de transmission bilatérale soit 6,6%. La TDM des rochers réalisée chez tous les enfants en préopératoire (Figure 3, Figure 4), avait montré un cholestéatome expansif avec comblement de toute la caisse du tympan, des cellules mastoïdiennes dans 20% des cas, un cholestéatome antro-attical chez 24 patients(80%). Une lyse ossiculaire a été mise en évidence chez 18 patients (60%). La lyse du mur de la logette chez 16 patients (53.3%), une lyse du tegmen tympani chez 4 patients (13.3%). Une lyse du canal facial dans 4 cas(13,3%). Aucune complications intracrâniennes n’ a été notée. Tous les enfants de notre série ont été opérés, avec en premier temps la réalisation d'une tympanoplastie en technique fermée, elle a consisté en une mastoïdectomie et tympanotomie postérieure avec conservation du mur du facial. La tympanoplastie en technique ouverte a été utilisée en cas de récidive après technique fermée dans 6 cas (20%). L’évolution à court terme a été en générale bonne et sans complication en postopératoire immédiat. L'audiogramme de contrôle a été réalisé chez tous les patients, à un mois, à trois mois et un an après l'intervention. On note un gain transmissionnel supérieur à20 db chez 10 cas (33,3%). Aucun cas d'aggravation ou de cophose iatrogène n'a été constaté. La TDM post-opératoire a été réalisée pour tous les patients dans un délai compris entre le 12 le 18^ème^ mois, on a noté la survenue de 8 cas de récidive (26,6%), tous réopérés par technique ouverte.

## Discussion

L'otite moyenne chronique cholestéatomateuse est une otite chronique évolutive, consécutive au développement dans l'oreille moyenne d'un épithélium malpighien kératinisé doté d'un potentiel de desquamation, de migration et d’érosion [1, 2], c'est une forme particulièrement agressive d'otite chronique, responsable de lésions ossiculaires, d'atteintes du nerf facial, de l'oreille interne, voire des structures cérébroméningées [3]. Les complications infectieuses, si elles ont longtemps été au premier plan avec les abcès cérébraux et les empyèmes, se résument maintenant le plus souvent à une infection chronique de l'oreille moyenne [3]. La physiopathologie du cholestéatome est encore mal comprise, on en distingue deux types: le cholestéatome congénital et le cholestéatome acquis [2, 4]. Pour ce dernier, l'hypothèse la plus fréquemment retenue est celle de l’évolution d'une poche de rétraction tympanique, avec apparition de lésions inflammatoires aigues et chroniques [4]. Plus rarement il peut apparaitre suite à la migration d’épithélium au travers d'une perforation marginale du tympan [4]. L’évolution du cholestèatome est sensiblement différente entre l'adulte et l'enfant, avec une agressivité particulière des cholestèatomes pédiatriques [5]. Cette évolution semble liée à une hyperexpression des metalloprotéases matricielles et a une angiogénèse accrue dans un contexte d'inflammation plus importante que chez l'adulte [4, 5]. Les signes cliniques évocateurs du cholestéatome sont l'otorrhée et l'hypoacousie. L'otorrhée est volontiers fétide, elle traduit le caractère surinfecté du cholestéatome [1, 2]. La surdité est habituellement transmissionnelle et correspond à la réaction inflammatoire ou à la destruction tympano-ossiculaire dans les formes évoluées [1, 2]. Les autres signes d'appels tels que les acouphènes, les otalgies et les vertiges semblent moins fréquents que chez l'adulte et surviennent surtout lors des phases de surinfection ou en présence de complications [1]. Les complications révélatrices tels que paralysie faciale, mastoïdite, labyrinthite, vertiges voire complications neuro-méningées ont beaucoup diminué en fréquence probablement en raison de la diffusion de l'antibiothérapie depuis plus d'un demi-siècle, ce quia a permis l'amélioration du pronostic vital des choléstèatomes de l'oreille moyenne [1, 4]. Le diagnostic positif de cholestèatome repose dans la majorité des cas sur le seul examen otoscopique.il doit être minutieux et réalisé Soit au microscope opératoire avec une aspiration contrôlée, soit sous contrôle otoendoscopique. Un bilan des deux oreilles s'impose. Plusieurs aspects otoscopiques peuvent être retrouvés: Le cholestéatome développé aux dépens de la pars tensa ou mesotympanique est plus fréquent chez l'enfant que chez l'adulte [4, 5], sous forme d'une masse blanchâtre nacrée correspondant à des débits épidermiques, la destruction ossiculaire est fréquente avec perforation tympanique marginale ou poche de rétraction. Le cholestèatome épitympanique ou attical pur visible derrière une perforation ou une poche de rétraction atticale [4, 5]. Le cholestéatome à tympan fermé, est rarement retrouvé, qu'il s'agisse d'une forme congénitale ou acquise, il se présente sous la forme d'une masse blanchâtre opaque bombante en arrière d'un tympan normal [6].

L'examen audiométrique fait partie intégrante du bilan d'un cholestéatome de l'oreille moyenne. Il existe habituellement une surdité de transmission due principalement à l'atteinte ossiculaire [4]. Il n'y a pas de corrélation systématique entre l'importance du Rinne et l'extension du choléstéatome. Il existe ainsi des choléstéatomes étendus avec un Rinne peu important [6]. La TDM est l'examen d'imagerie de référence pour l’étude de l'oreille Moyenne [7]. Dans l'otite chronique cholestéatomateuse, elle permet d'une part d'effectuer un bilan lésionnel précis, elle permet de préciser le siège, l'extension et les conditions anatomiques de l'intervention, chirurgicale et d'autre part d'aider au diagnostic lorsque l'examen clinique est douteux [3, 7]. Aussi elle est devenue performante dans le dépistage des récidives et des résiduels du cholestéatome dans le cadre de surveillance post-opératoire [3, 7]. Les deux signes tomodensitométriques principaux en faveur du diagnostic de cholestéatome sont une masse de densité tissulaire des cavités tympano-mastoïdiennes et une ostéolyse des éléments ossiculaires, ces deux signes sont le plus souvent associés [7]. Le volume et la pneumatisation des cavités tympano-mastoïdiennes doivent être pris en considération sur le scanner pré-opératoire, et peuvent influer sur le choix de la technique chirurgicale [2, 7]. L'IRM est peu pratiquée dans le cadre du bilan d'extension initial, sauf en cas de suspicion de complications endocrâniennes, elle reste cependant indiquée pour la surveillance postopératoire, en cas de d'images douteuses à l'examen TDM [4, 5]. Le traitement de l'otite moyenne chronique choléstèatomateuse reste exclusivement chirurgical, visant l’éradication de la maladie afin d'obtenir une oreille saine et aérée, mais également le rétablissement ou l'amélioration de l'audition [6, 8]. Le choix entre tympanoplastie en technique fermée et tympanoplastie en technique ouverte, dépend de nombreux paramètres: l’état de l'oreille malade et de l'oreille controlatérale, l'audition, les antécédents otologiques et généraux, le terrain naso-sinusien, la tomodensitométrie pré-opératoire [9]. De nombreuses équipes privilégient la tympanoplastie en technique fermée chez l'enfant, la nécessité d'un deuxième temps opératoire ou second look doit être déterminée en fonction des critères radiologiques préopératoires et les découvertes opératoires [4, 9]. La généralisation de l'utilisation du cartilage comme matériau de reconstruction du cadre et du tympan a entraîné une réduction significative du taux de récidive cholestéatomateuse [9]. La surveillance a long terme doit faire partie intégrante de la prise en charge thérapeutique quelque soit la technique choisie, elle permet de dépister les cholestèatomes résiduels et les récidives, qui semblent être plus fréquentes chez les enfants que chez les adultes, témoignent de l'agressivité particulière du cholestéatome de l'enfant [3, 5].

## Conclusion

La pathologie cholestéatomateuse est potentiellement dangereuse chez l'enfant par sa tendance destructrice et récidivante, car elle survient sur un terrain de dysfonction tubaire lui conférant une agressivité et un potentiel invasif supérieur. Son diagnostic doit être précoce, la TDM reste l'examen de choix pour le bilan d'extension préopératoire. Le traitement du cholestéatome est exclusivement chirurgical et la technique fermée reste préférée chez l'enfant.
